# Delayed Intramural Duodenal Hematoma After a Simple Diagnostic Endoscopic Ultrasonography Fine-Needle Aspiration Procedure

**DOI:** 10.14309/crj.0000000000000279

**Published:** 2019-11-25

**Authors:** Joana Roseira, Miguel Cunha, Helena Tavares de Sousa, Juan Rachadell, Jorge Brito

**Affiliations:** 1 Gastroenterology Department, Algarve University Hospital Center, Portimão, Portugal; 2 Algarve Biomedical Center, University of the Algarve, Portimão, Portugal; 3 General Surgery Department, Algarve University Hospital Center, Portimão, Portugal; 4 Radiology Department, Algarve University Hospital Center, Portimão, Portugal

## CASE REPORT

**Figure 1.**
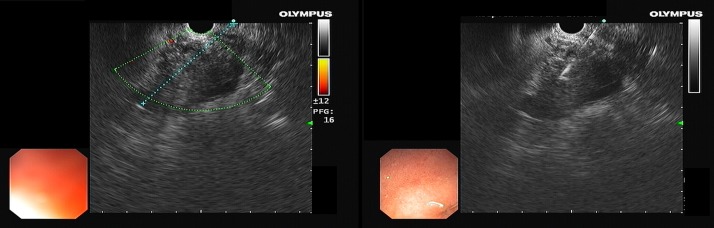
Endoscopic ultrasound-guided fine-needle aspiration procedure.

**Figure 2.**
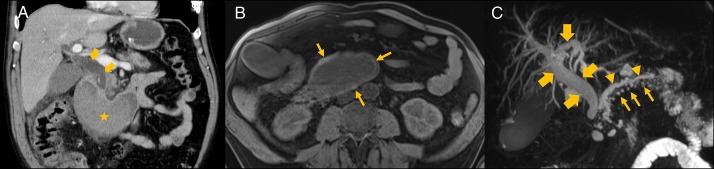
(A) Abdominal computed tomography showing an intramural mass (star) extending from the duodenal knee to the third portion, compressing the duodenal lumen with biliary duct dilatation (arrows). (B) Fat-suppressed T1-weighted axial image reveals textural heterogeneity with hyperintense focuses (arrows) in the periphery of the lesion. (C) Fat-suppressed T2-weighted coronal 3D sequence showing biliary tree dilatation (thick arrows) with irregular dilatation of the main pancreatic duct (arrowheads) and ectatic secondary branches (thin arrows).

A 65-year-old man was evaluated for a difficult-to-characterize pancreatic head mass in the setting of idiopathic chronic pancreatitis. He had no other relevant medical history and was not taking any anticoagulant or antiplatelet treatment. Endoscopic ultrasonography fine-needle aspiration (EUS-FNA) failed to reveal neoplasm cells. A linear array echoendoscope (Olympus GF-UCT140, Center Valley, PA) was advanced up to the duodenal bulb, from which the head of the pancreas was visualized. After ensuring a vessel-free access to the pancreatic parenchyma, the FNA was performed using a 22G needle (Slimline 22G Handle Needle; Boston Scientific, Marlborough, MA) with a total of 3 passes (Figure 1). Three weeks after this procedure, the patient was admitted for hematemesis preceded by vomiting. On admission, his general physical examination was unremarkable except for jaundice. His blood tests showed no anemia; his platelet count, prothrombin time, amylase, and liver enzymes were within normal range, but his total bilirubin level was elevated (7.4 mg/dL). Upper gastrointestinal endoscopy showed Mallory-Weiss tears, an evident extrinsic compression of the knee, and the second portion of the duodenum, which could not be passed by the endoscope. The investigation by computed tomography and magnetic resonance cholangiopancreatography led to the diagnosis of an 11-cm intramural duodenal hematoma (IDH), leading to both gastric outlet and main biliary duct obstruction (Figure 2). The case was successfully managed with nasogastric decompression and exclusive parenteral feeding. Symptoms improved within 15 days, and cholestasis progressively disappeared.

IDH caused by mechanisms other than blunt abdominal trauma or nonaccidental injury to children, namely concerning child abuse, are rare.^1,2^ The American Society for Gastrointestinal Endoscopy guidelines on EUS-FNA adverse events report an overall bleeding rate of 0.13% from a recent meta-analysis, and IDH does not figure in these complication lists.^3,4^ Recently, a report of an IDH after EUS-guided implantation of pancreatic cancer fiducials was published.^5^ Similarly, we hypothesize that a combination of endoscope torqueing, manipulation of the duodenal mucosa, and prolonged procedure time may have contributed for this complication. This patient was hemodynamically stable; there were no signs of complications (anemia, hematoma perforation, or pancreatitis), and there were no imaging traces of active hemorrhage nor luminal bleeding. In addition, because endoscopic retrograde cholangiopancreatography was not feasible owing to inaccessible duodenal papillae, he was conservatively managed with success, as currently proposed.^1^ The rational for a nonsurgical approach in stable patients is related to an expected spontaneous reabsorption of the hematoma because of the abundant irrigation of the area. This report points out an exceptional case of a simple EUS-FNA diagnostic procedure, complicated by a voluminous IDH, presenting with gastric outlet obstruction, upper gastrointestinal bleeding related to Mallory-Weiss tears, and important cholestasis.

## DISCLOSURES

Author contributions: J. Roseira wrote the manuscript. M. Cunha and J. Rachadell managed the patient. HT de Sousa and J. Brito revised the manuscript. J. Brito is the article guarantor.

Financial disclosure: None to report.

Informed consent was obtained for this case report.

Previous presentation: This case was presented at Portuguese Digestive Disease Week, May 9–June 1, 2019; Algarve, Portugal.
